# Mechanisms of microbial co-aggregation in mixed anaerobic cultures

**DOI:** 10.1007/s00253-024-13246-8

**Published:** 2024-07-04

**Authors:** Anna Doloman, Diana Z. Sousa

**Affiliations:** 1 Laboratory of Microbiology, Wageningen University & Research, Stippeneng 4, 6708 WE Wageningen, The Netherlands; 2 Centre for Living Technologies, Eindhoven-Wageningen-Utrecht Alliance, Princetonlaan 6, 3584 CB Utrecht, The Netherlands

**Keywords:** Anaerobic biofilm, Co-aggregation, Exopolysaccharides, Syntrophy, Cell-to-cell adhesion

## Abstract

**Abstract:**

Co-aggregation of anaerobic microorganisms into suspended microbial biofilms (aggregates) serves ecological and biotechnological functions. Tightly packed aggregates of metabolically interdependent bacteria and archaea play key roles in cycling of carbon and nitrogen. Additionally, in biotechnological applications, such as wastewater treatment, microbial aggregates provide a complete metabolic network to convert complex organic material. Currently, experimental data explaining the mechanisms behind microbial co-aggregation in anoxic environments is scarce and scattered across the literature. To what extent does this process resemble co-aggregation in aerobic environments? Does the limited availability of terminal electron acceptors drive mutualistic microbial relationships, contrary to the commensal relationships observed in oxygen-rich environments? And do co-aggregating bacteria and archaea, which depend on each other to harvest the bare minimum Gibbs energy from energy-poor substrates, use similar cellular mechanisms as those used by pathogenic bacteria that form biofilms? Here, we provide an overview of the current understanding of why and how mixed anaerobic microbial communities co-aggregate and discuss potential future scientific advancements that could improve the study of anaerobic suspended aggregates.

**Key points:**

*• Metabolic dependency promotes aggregation of anaerobic bacteria and archaea*

*• Flagella, pili, and adhesins play a role in the formation of anaerobic aggregates*

*• Cyclic di-GMP/AMP signaling may trigger the polysaccharides production in anaerobes*

## Introduction

**Fig. 1:**
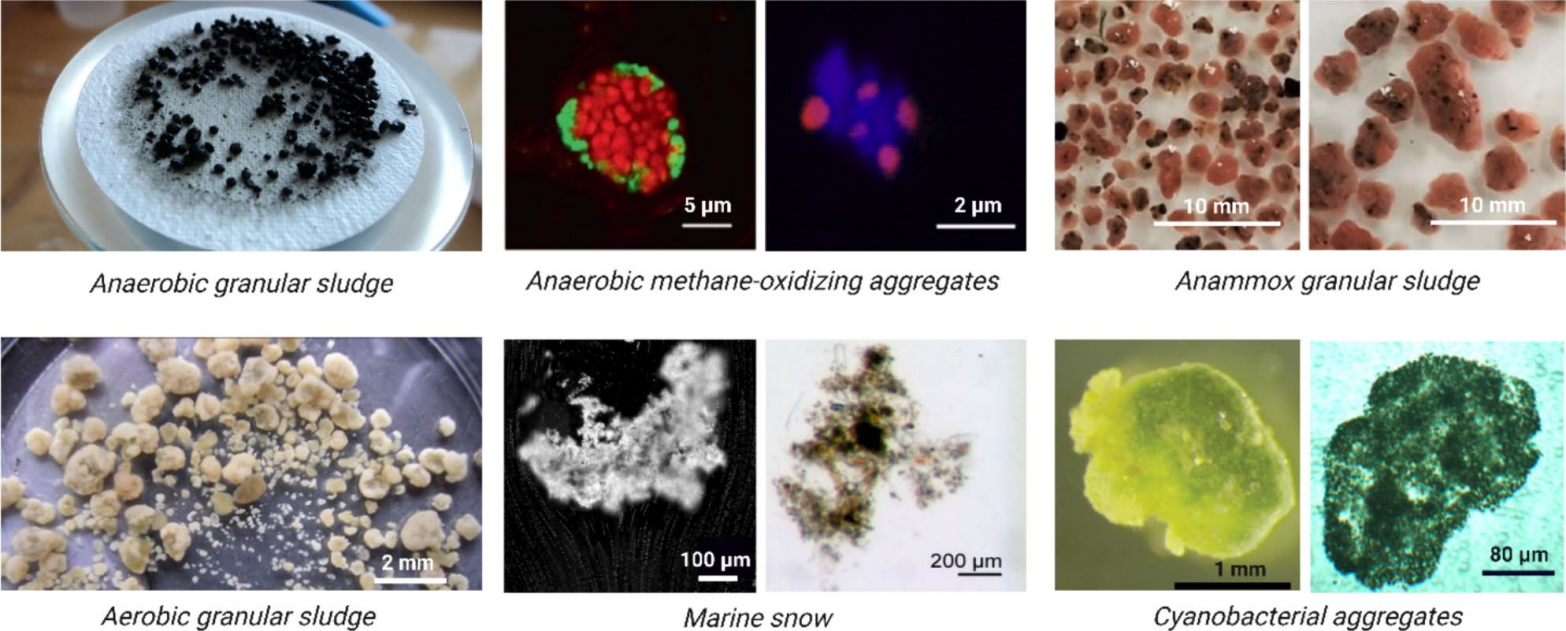
Examples of microbial aggregates in anoxic (upper pane) and oxic/microaerophilic environments (bottom pane). Images of anaerobic methane-oxidizing aggregates are confocal laser scanning micrographs depicting hybridization with the fluorescent probes for archaea (red or pink) and bacteria (green), while blue color depicts general DNA stain. Images of aggregates reproduced with permission (Knittel and Boetius 2010; Jagersma et al. 2012; Gonzalez-Gil and Holliger 2014; Laurenceau-Cornec et al. 2015; Wilbanks et al. 2017; Lin et al. 2022; Chajwa et al. 2023)

Most of the prokaryotic life on Earth lives in biofilms. Microbial biofilms of various shapes and forms are omnipresent in natural and engineered ecosystems in either surface attached or suspended configurations. *Surface-attached biofilms* cover soil particles, water–air interfaces, plant roots and leaves, as well as guts of insects, animals and humans (Flemming and Wuertz 2019). Meanwhile, *suspended biofilms (aggregates)* are more often found in engineered environments, such as wastewater-treating bioreactors and food fermenting facilities (Grotenhuis 1992; Gonzalez-Gil et al. 2001; Feng et al. 2024). Naturally occurring suspended biofilms can be also found in the form of marine and freshwater “snow” and cyanobacterial surface blooms (Jankowiak and Gobler 2020; Li et al. 2021) (Fig. 1). Within biofilms, diverse species of microorganisms are exchanging phosphorus, nitrogen and carbon-containing nutrients. In addition, microorganisms may utilize the biofilm matrix as a shield, offering protection against, e.g., environmental stressors or antimicrobial agents.

Historically, most of the research on biofilms has centered on disease-associated aerobic or aerotolerant microorganisms, commonly growing as surface-attached biofilms within the mucosal layers of their eukaryotic hosts (Sauer et al. 2022). The widespread application of molecular techniques, such as amplicon and metagenome sequencing, allowed to broaden the areas of biofilm research to environmental biofilms. Recent reviews offer up-to-date comprehensive insights and perspectives into the study of surface-attached environmental biofilms and microbial interactions within them (Flemming and Wuertz 2019; Sadiq et al. 2022; Qian et al. 2022). Furthermore, the research into suspended biofilms, both in nature and in biotechnological processes, represents a rapidly evolving field (Cai 2020; Kragh et al. 2023). In addition to aerobic suspended biofilms, such as those found in activated sludge systems used for the oxygen-intensive treatment of municipal wastewater, there is a growing interest in *anaerobic aggregates*, commonly referred to as “anaerobic granules” (Trego et al. 2021; Mills et al. 2024). There are several examples of anaerobic granules, all harboring mixed communities of bacteria and/or archaea, involved in, e.g., conversion of complex organic materials during the anaerobic digestion of wastes and wastewaters, anaerobic ammonium oxidation (anammox process), or even anaerobic methane oxidation (Fig. 1).

This mini review bridges the knowledge gap between the surface-attached (an)aerobic biofilms and suspended anaerobic aggregates, to provide a common ground for discussion in the scientific field. Specifically, *the review addresses the progress in understanding of the mechanisms of microbial interactions that lead to the formation of multispecies anaerobic bacteria-archaea aggregates.* Therefore, the reader can expect analogies with the more advanced field of surface-attached biofilms and host-associated aerobic aggregates (Hede and Khandeparker 2020; Bridges et al. 2022; Flemming et al. 2023). The review also includes a summary of the future directions in the field that will allow to study in detail the high diversity of the anaerobic bacteria-archaea aggregates.

## Structural diversity of the multispecies anaerobic aggregates and methods for research on co-aggregation

As in the few other studies of microbial suspended biofilm formation, the term “co-aggregation” refers to the adherence of different microbial species to each other, resulting in the formation of spherical or irregular-shaped aggregates. Mixed anaerobic aggregates show high functional and morphological diversity. Early studies have used a variety of techniques to investigate this diversity, with specific focus on studying the microbiological composition of mature mixed culture aggregates. Invaluable insights were observed on the structure and composition of aggregates by performing scanning and transmission electron microscopy, and confocal microscopy often assisted with fluorescent labelling of the microorganisms of interest (either with general archaea/bacteria probes, or with specific genus/species probes) (Zheng et al. 2006; Knittel and Boetius 2010; Wang et al. 2020). These observations revealed a wide array of aggregate architectures, with microbial groups either organized in tightly packed clusters or shuffled within the aggregates’ layers.

Layered distribution of microorganisms in granules from biotechnology industry, like aerated wastewater treatment basins, phototrophic bioreactors, and anaerobic digesters, allows for the effective transformation of organic matter. Outer layers of both aerobic and phototrophic granules are populated by oxygen-reducing or photosynthesizing microorganisms like algae, cyanobacteria, and some (de)nitrifying bacteria (e.g., *Nitrosomonas*, *Thauera*) (Milferstedt et al. 2017; Cydzik-Kwiatkowska et al. 2022; Trebuch et al. 2023). The core of both photo- and aerobic granules is more anoxic and is thus populated by anaerobic fermenting bacteria, like members of *Anaerolineaceae.* As a result of such arrangement, photogranules can simultaneously achieve fixation of carbon, production of nitrate, and even sugar fermentation. The latter occurs in the inner layers and can be used to (re)supply nutrients to the outer layers in case of starvation in the photoautotrophic zones of the granule. Aerobic granules can achieve similar carbon and nitrogen transformations as photogranules, as well as recycling of phosphorous (Weissbrodt et al. 2013).

Anaerobic granules, such as the ones found in anaerobic digesters, are generally also organized in layers. There, bacteria carrying primary fermentations or acetogenesis are often found either on the outside layer of the aggregate or mixed throughout its depth, depending on the kinetics of primary substrate hydrolysis in the bioreactor (Lu 2014). Microbial groups that are not responsible for the initial hydrolysis of the substrates, or that are more sensitive to oxygen, like strictly anaerobic methanogenic archaea and fatty-acid degrading bacteria (e.g., *Methanobacteriaceae* and *Syntrophobacter*), are more commonly found in the middle layers of the aggregates (Tsushima et al. 2010; Lu 2014). On the contrary, granules in anammox (anaerobic ammonium oxidation) bioreactors rarely have such layered structure. Instead, these granules are comprised of small sub-clusters of ammonium-oxidizing bacteria (e.g., “*Candidatus* Brocadia”) embedded within the network of filamentous fermenting *Anaerolinea* and a mixture of bacteria performing heterotrophic denitrification (Gonzalez-Gil et al. 2015; Li Wong et al. 2023). Aggregates performing anaerobic methane oxidation found in methane-rich marine environments are comprised of anaerobic methanotrophic archaea (ANME) in the core, surrounded by the layer of diverse sulfate-reducing bacteria (Knittel et al. 2005; McGlynn et al. 2018; Murali et al. 2023).

After the discovery of the microbial and structural diversity of anaerobic aggregates, several research groups attempted to isolate microbial representatives that are key to the transformations within these aggregates. However, many of the isolation attempts failed to obtain pure cultures of these microorganisms, instead enriching for the metabolically dependent co-cultures. Such was the discovery of obligate syntrophic co-cultures comprised of fatty acid–oxidizing bacteria and hydrogenotrophic methanogenic archaea (Sousa et al. 2009; Stams and Plugge 2009). Examples of such partnerships include pairing between *Syntrophobacter fumaroxidans* and *Methanobacterium formicicum*, or between *Syntrophomonas wolfei* and *Methanospirillum hungatei* (McInerney et al. 1981; Harmsen et al. 1998). The oxidation of fatty acids by the bacterium becomes exergonic only when H_2_/formate levels are kept low, which is facilitated by the methanogens capable of converting H_2_/formate to methane. Some syntrophic bacteria were found to be able to grow axenically on fumarate or crotonate, when no methanogenic partner is available. However, these intermediates are not found in the anaerobic digestion systems, thus being metabolic detours created purely for the laboratory purposes to allow physiological studies of these microorganisms.

Isolation attempts from anammox and anaerobic methane oxidizing aggregates were less fruitful. To date, there are no pure cultures of either “*Candidatus* Brocadia” (member of anammox) or “*Candidatus* Methanoperedens” (member of ANME), pointing to the extremely strict nature of the microbial symbioses they engage in (Lu et al. 2022; Ouboter et al. 2024). Research into the use of alternative non-bacterial electron acceptors, like insoluble iron oxides as terminal electron acceptors for denitrifying anaerobic methane-oxidizing archaea, might hold the key to the future successful pure culture isolation attempts (Bhattarai et al. 2019). For now, highly enriched bioreactor cultures of these microorganisms can be used to gain valuable insights into their physiology.

Application of cultivation-independent (meta-omics) techniques to analyze the activity within the granular anaerobic microbial communities is opening doors for in-depth studies overcoming pure culture isolation hurdles. A successful example is the use of metatranscriptomics to study the activity of microbial communities during the aggregation process. Observations from gene expression profiles in aerobic bacterial aggregates were found to mirror those in the studies of microbial activity in anaerobic microbial communities (Bagchi et al. 2016; Aqeel et al. 2019; Kragh et al. 2023; Doloman et al. 2024a). Specifically, microorganisms in both aerobic and anaerobic aggregates had upregulated expression of signal transduction gene circuits and of genes responsible for the secretion of extracellular biofilm components, compared to the non-aggregated dispersed microorganisms. The upregulation of genes responsible for the production of extracellular polymeric substances (EPS) stabilizes the strength of cell–cell interactions in mature aerotolerant host-associated bacterial aggregates of *E. coli* or *Salmonella* spp. with *Vibrio* spp*.*, *Streptococcus* spp., or with *Actinomyces* spp. (Rickard et al. 2006; Elias and Banin 2012; Mutha et al. 2019). A similar result is expected from the increased expression of EPS biosynthesis clusters in anaerobic aggregates of sulfate-reducing or syntrophic fatty acid-oxidizing bacteria (Mao et al. 2015; Doloman et al. 2024a). However, the mechanisms of the initial cell-to-cell contact-dependent co-aggregation in anaerobic mixed cultures are hypothesized to differ from the mechanisms of the surface-attached biofilm formation in the aerotolerant bacteria (Cai 2020; Sauer et al. 2022). In particular, is mechanosensing machinery of surface-attaching bacteria also involved in sensing the surface of other cells in the surface-free co-aggregation? Since the energy available for growth of anaerobic bacteria is typically 10 × lower than that for aerobic bacteria, do the former express less cellular appendages in their life cycle and cut down resource use when transitioning into the aggregated growth stage? We will now look into these parallels and summarize the available knowledge on the microbial drivers of surface-free co-aggregation of anaerobic co-cultures occurring in natural and engineered environments.

## Reasons for anaerobic microbial co-aggregation—for food and shelter

To start understanding the mechanisms of anaerobic microbial co-aggregation, it is important to identify the reasons for such behavior. Many aerobic microorganisms that have been observed to aggregate do so under stress, such as during limited availability of oxygen or nutrients, upon changes in pH, salinity or temperature, or in the presence of calcium or magnesium (Trunk et al. 2018; Nwoko and Okeke 2021). Aggregation may be also affected by the cell population density, ratio of the co-aggregating partners and their nutritional dependencies, as well as the number of microorganisms that are able to produce EPS that facilitate cell-to-cell adhesion (Doloman et al. 2020). The physical forces governing the mixed microbial aggregation, such as of cell-to-cell electrostatic attraction, hydrophobicity of the cell surface–promoting cell interaction, and eventual co-aggregation has been well analyzed and reviewed for both aerobic and anaerobic mixed-culture aggregates (Yuan et al. 2018)*.* What is yet missing in the well-organized form is the knowledge on the unique role of microbial metabolic interdependencies and cell-associated appendages (flagella, pili) in facilitating the formation of suspended aggregates.

### A nutritional need to cooperate

From the studies of single species bacterial aggregation, it was proposed that formation of aggregates is promoting harsh competition for resources, since cells located on the outside of the aggregates have access to higher concentrations of substrates/electron acceptors (Trunk et al. 2018). On the other hand, arrangement of diverse microbial strains into a biofilm shortens the cell–cell distances, thus promoting exchange of metabolites and giving rise to the synergistic relationships (Dal Co et al. 2020; Kost et al. 2023). Therefore, biofilms are greatly beneficial for auxotrophic microorganisms, or microorganisms that will otherwise not survive in nutrient-poor oligotrophic environments (Zengler and Zaramela 2018; Yin et al. 2019). Interestingly, in silico simulations of growth and metabolite exchange within a facultatively anaerobic microbial community of 14 species showed that absence of oxygen promoted mutualistic cooperations (4 × more than in the simulations with oxygen) (Pacheco et al. 2019). The closest proxy to support these modelling observations can be derived from observing the nature of microbial interactions in mixed-culture aggregates of aerobic and anaerobic (waste)water treatment facilities and fresh water/marine environments. While both aerobic and anaerobic aggregates have a rich diversity of cooperative relationships, presence of diverse terminal electron acceptors (oxygen, nitrate, sulfate, CO_2_) merely causes a stratification of microorganisms within the aggregates along the gradient of oxygen succession, with predominantly commensal and competitive metabolite exchange (Xavier et al. 2007). Absence of oxygen, on the contrary, promotes a cooperative microbial division of labor and distribution of microorganisms along the gradient of metabolites that are exchanged mutually beneficially. In such way, complex organic molecules are effectively transformed/degraded as a result of cooperation of multiple species (and presence of multiple enzymatic machineries) (Doloman et al. 2017, 2020). Division of labor can also lead to the niche clustering of cooperating species around the primary fermenting species (Moons et al. 2009; Micali et al. 2023). In this way, products of metabolism of one species are used as substrates for growth by another species, resulting in the conversion of complex organic molecules. Embedding of the microorganisms in a shared EPS matrix further facilitates the flow of the metabolites, increasing the bioavailability of the carbon/energy sources for the whole aggregated community. As a result, spatial microbial arrangement in the aggregates allows cross-feeding communities to maintain effective metabolic interactions and continue growing in the environments with intermittent nutrient availabilities (Micali et al. 2023).

For example, the efficiency of metabolic conversions within the industrial wastewater treating granules is fully dependent on the microbial metabolic interactions and interdependencies. These interdependencies are supported by layered arrangement of microorganisms within the aggregates (e.g., aerobic, anaerobic, and phototrophic granules) along the gradient of (**a**) electron acceptors, like oxygen/nitrogen oxides (anoxic zones within the aggregates are occupied by the strict anaerobes); (**b**) electron donors, substrates (e.g., fermenting bacteria in the outer layers, and acetate/hydrogen/formate-oxidizing bacteria and methane-producing archaea in the inner cores of the anaerobic aggregates) (Gonzalez-Gil et al. 2001; Xavier et al. 2007; Doloman et al. 2017, 2020).

**Fig. 2:**
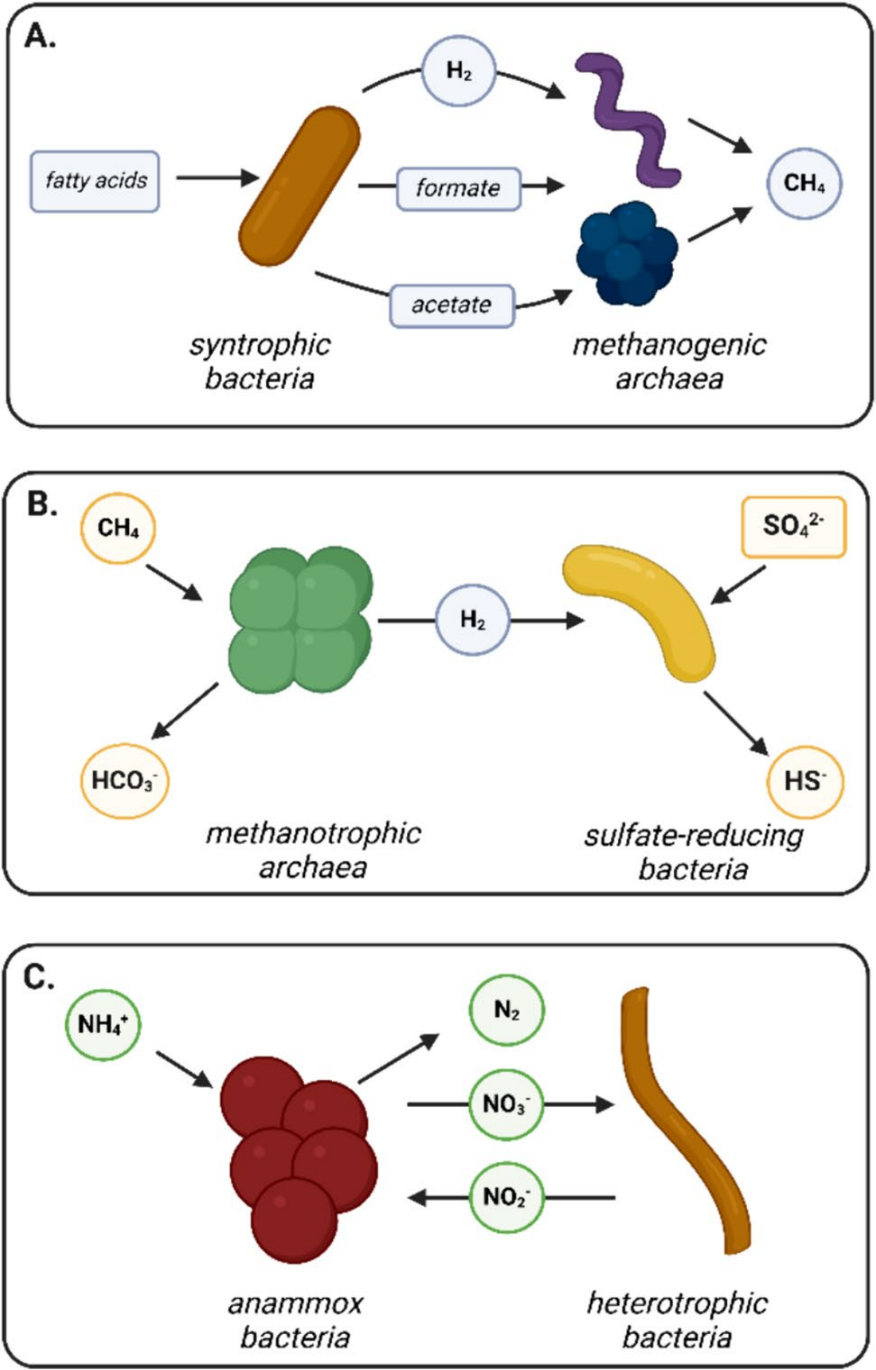
Key microbial symbiotic relationships within **A** anaerobic granules from anaerobic digesters, **B** anaerobic methane oxidation aggregates, and **C** anaerobic ammonia oxidation aggregates. Illustration was created with BioRender.com

Co-aggregation is also the preferred growth mode for the bacteria and archaea involved in obligate syntrophy (“eating together”), a case of strict metabolic interdependency (Ishii et al. 2005; Stams and Plugge 2009; Doloman et al. 2024a), originally discovered for hydrogen- and formate-exchanging microbial communities in the anaerobic granular sludge (Fig. 2A). Such exchange allows for the otherwise thermodynamically challenging oxidation of volatile fatty acids (VFAs) such as acetate, propionate, and butyrate into methane. As a result of hydrogen/formate shuttling, the syntrophic bacteria (e.g., *Syntrophomonas*, *Syntrophobacter*) and archaea (hydrogenotrophic methanogenic archaea) generate and share ~ 76 kJ/mol of substrate. Studies of long-term laboratory fed-batch cultivations of syntrophic propionate- or butyrate-oxidizing co-cultures demonstrated preference of both bacteria and archaea partners to co-exist in a tightly arranged aggregated mode (Mollaei et al. 2021; Doloman et al. 2022, 2024a). In these examples, aggregated growth mode was found to be beneficial for both involved partners, leading to the improved substrate oxidation and methane generation rates, as well as decreased the lag phase upon transfer of the cultures into the new media (Doloman et al. 2022; Besteman et al. 2024).

Syntrophy and microbial interdependencies also drive co-aggregation of marine anaerobic microorganisms, such as methanotrophic archaea and sulfate-reducing bacteria (e.g., *Desulfosarcina*/*Desulfococcus*) (Ruff et al. 2013). Due to the hydrogen exchange during the syntrophic methane oxidation, sufficient Gibbs energy is conserved to allow growth of these two partnering microorganisms (~ 40 kJ/mol CH_4_) (Fig. 2B). Co-aggregation of fresh water–dwelling anaerobic ammonium–oxidizing bacteria and their denitrifying partners is also a result of metabolic interdependency (Wang et al. 2020) (Fig. 2C). In these aggregates, denitrifying bacteria reduce nitrate to nitrite, and nitrite is used as an electron acceptor by the anammox bacteria to oxidize ammonium to dinitrogen gas. Moreover, bacteria inside the aggregates exhibit functional complementarity in nitrogen and amino acid metabolism, and exchange secondary metabolites such as molybdopterin cofactor, folate, and nucleotide sugars.

Non-syntrophic but electron-shuttling communities of some methanogenic archaea (*Methanosaeta*, *Methanosarcina,* some *Methanobacterium*) and electrogenic bacteria (*Geobacter*) have also shown tendency to grow in the aggregated mode (Rotaru et al. 2014; Yee and Rotaru 2020; Zheng et al. 2020). In those aggregates, *Geobacter* is using the methanogen partner as an electron acceptor during the oxidation of ethanol. A similar co-aggregation pattern was also observed for ethanol oxidizing sulfate-reducing *Desulfovibrio* spp., which formed aggregates with electrons accepting *Methanosaeta* or *Methanobacterium* (Zheng et al. 2021). Recent review of the genetic make-up of the 46 sulfate-reducers often found in the methane-oxidizing microbial aggregates suggests that extracellular electron shuttling is also important for the establishment of the syntrophic associations between anaerobic methane-oxidizing archaea and sulfate-reducing bacteria. Majority of the described cases of proposed electron shuttling are hypothesized to occur via multiheme cytochrome:porin type conduits, previously found to be common for the known exoelectrogen *Geobacter* and widespread among other Gram-negative bacteria (Murali et al. 2023).

### Some just like to “stick together”

Although metabolically dependent groups of microorganisms have a clear advantage to reside in tight aggregates where they have fast access to the partner-produced resources, a few microorganisms play bridging or matrix building role. In environments like freshwater, activated sludge wastewater–treating bioreactors, and oral biofilm–forming microorganisms, bridging microbial species promote co-aggregation of normally non-aggregating species. Examples of such bacteria include *Fusobacterium, Blastomonas, Micrococcus, Methylobacterium, Sphingomonas*, and *Acinetobacter* (Rickard et al. 2002, 2003; Katharios-Lanwermeyer et al. 2014; Stevens et al. 2015). Some of these bacteria (like *Acinetobacter johnsonii*) were shown to be highly hydrophobic and with a slightly negative surface charge, which would explain the electrostatic attraction to cells with more positively charged cell surfaces (Malik et al. 2003). Other bridging bacteria were found to have a number of cell surface–associated adhesins, 70–300 kDa proteins containing repeated amino acid sequences, located at the tips of the bacterial fimbria or along the cell membrane. To facilitate co-aggregation, cell surface adhesins bind to the saccharide-containing receptor molecules on the surface of the other cells. However, little is known on the exact mechanisms of adhesin-receptor co-aggregation beyond studies on streptococci (Yoshida et al. 2014; Afonso et al. 2021).

While identities and characteristics of the strictly anaerobic bridging microorganisms remain to be revealed, there are a few possible candidates. In general, microorganisms isolated from anaerobic granular sludges were found to be more hydrophobic compared to the facultatively anaerobic microorganisms or the anaerobic ones living in suspensions (Grotenhuis 1992; Daffonchio et al. 1995). Examples of such highly hydrophobic microorganisms are methanogenic archaea from genera like *Methanobrevibacter*, *Methanosaeta*, and *Methanosarcina.* Although not much research has been done on this subject in the last 30 years, all these methanogens are repeatedly found throughout industrial- or laboratory-sourced granular anaerobic sludges, supporting the importance of methanogens for the stability of the mixed anaerobic aggregates (Zheng et al. 2006; Trego et al. 2020; Doloman et al. 2024b). Recent work with tri-cultures comprised of butyrate-oxidizing *Syntrophomonas wolfei*, hydrogenotrophic methanogens (*Methanobrevibacter arboriphilus*, *Methanobacterium formicicum*, *Methanospirillum hungatei*), and glycerol-degrading *Trichococcus flocculiformis* demonstrated the possibly bridging role of the later (Doloman et al. 2022). However, the mechanism of the *Trichococcus*-bridging behavior remains to be elucidated.

## The stages of anaerobic co-aggregation

Currently, there is no unified mechanistic model that could explain aggregate formation in multi-species microbial communities, especially if they involve mixed bacteria–archaea populations. There have been some very good attempts to describe aggregation phenomenon conceptually (Cai 2020; Sauer et al. 2022; Kragh et al. 2023) and even model the 3D arrangement of the cells based on the microbial kinetics of substrate/product turn-over (Xavier et al. 2007; Doloman et al. 2017, 2020). While some physiological data associated with co-aggregation is available for the granular sludge and environmental aggregates (Afonso et al. 2021; McIlroy et al. 2023; Feng et al. 2024), in-depth time-resolved gene, and protein regulation studies have only been made for the aggregate formation in pathogenic microbial communities (Bagchi et al. 2016; Livingston et al. 2022; Manner et al. 2023; Condinho et al. 2023). In those communities, co-aggregation was suggested to occur between two or more genetically distinct strains, which interact by specific cell–cell recognition (Choo et al. 2021). Since majority of the cells in mixed environmental aggregates and industrial granules are indeed composed of genetically distinct populations, it is possible that these communities use similar cell–cell recognition mechanisms as the pathogenic bacteria in surface-non-attached biofilms. Such similarity could explain the one feature universally present in all the biofilms, attached or suspended, which is the presence of EPS. EPS are embedding/surrounding microorganisms and ultimately play the role of protecting the aggregated microbial population from the negative influence of the fluctuating environmental factors (pH, ionic strength, hydrodynamic shear force, dissolved oxygen content) (Flemming et al. 2023). Based on these assumptions, we can define two general steps in the co-aggregation process: (1) partner recognition as the aggregate initiation step; (2) regulation of EPS production as the aggregate maturation step.

### Partner recognition as the aggregate initiation step

Attached biofilms are initiated by mechanosensing of the surface by the bacterial flagella and the intracellular regulatory cascade, fairly well-known for many pathogenic bacteria (Belas 2014). Cell surface proteins of these bacteria (adhesins, flagella, pili, fimbriae), exopolysaccharides (ex. poly-N-acetylglucosamine), or even extracellular DNA have all been shown to mediate the initial cell–cell contact (Trunk et al. 2018). Recent review of bacterial biofilm formation in various aquatic environments also addressed the importance of cell surface–associated molecules, like adhesins, in promoting cell partner recognition and cellular attachment to the surface substrata (Afonso et al. 2021). A similar system might be involved in the cell–cell recognition and initial adhesion that is required for the initiation of the aggregate formation in non-surface associated environmental and biotechnology-relevant bacteria-archaea co-cultures.

#### Adhesins

Genome-wide surveys reveal abundance of amyloid adhesins encoded in the genomes of representatives of various bacterial phyla, like Proteobacteria, Bacteroidetes, Firmicutes, and Thermodesulfobacteria (Aqeel et al. 2019). These protein-rich fibrous structures were found to cluster together in the extracellular space and form rope-like arrangements surrounding cells in the mature aggregates. Carbohydrate-binding counterparts of adhesins, lectins, were found to also play a critical role in stabilizing the cell–cell interactions and are widespread in both aerobic and anaerobic aggregates (Gagliano et al. 2018; Neu and Kuhlicke 2022). It was found that amyloid adhesins constitute a relatively large fraction of EPS in the activated sludge aggregates and aerobic granular sludge (Larsen et al. 2008; Lin et al. 2018). Specific amyloid adhesins antibodies were found to bind to the abundant in activated sludge denitrifiers, like *Thauera*, *Zoogloea*, and *Azoarcus*, as well as to some of the filamentous *Actinobacteria*, *Aquaspirillum*, and *Chloroflexi*. Meanwhile, probiotic bacteria (e.g., *Lactobacillus*) have been shown to poses mannose-specific adhesin that recognizes either the surface layer proteins (S-layer proteins) of the co-aggregating partners (e.g., *S. cerevisiae*), or the mucus layer of the host intestinal tract (Pretzer et al. 2005). Recent studies on co-aggregation of propionate-oxidizing *S. fumaroxidans* and methanogens*, M. hungatei* or *M. formicicum,* revealed a significant overexpression of the bacterial fibronectin type III proteins and putative outer membrane adhesin-like proteins in the year-old aggregates (Doloman et al. 2024a). In those aggregates, the archaeal partner, *M. formicicum*, had a highly expressed OmcB-like cysteine-rich periplasmic protein with conserved DUF11 domain, which is hypothesized to play a key role as a membrane-bound adhesion protein (Sumikawa et al. 2019). The only adhesin-like protein found overexpressed in *M. hungatei* (when co-aggregating with *S. fumaroxidans*) was the major sheath protein MspA, which can assemble into amyloid assemblies that are hypothesized to be involved in maintaining cell–cell adhesion (Christensen et al. 2018). Amyloidogenic nature of MspA was originally confirmed for another S-layered methanogen, *Methanosaeta thermophila*, which compared to *M. hungatei*, forms much longer multi-cell filaments (Dueholm et al. 2015). Other studies on archaeal adhesins are scarce and have not been focused yet on the co-aggregation with bacteria. Among self-aggregating archaea, *Methanothermobacter thermoautotrophicus* was the first to be shown to possess a 16-kDa fimbrial glycoprotein that acted as an adhesins by mediating both intraspecies cell–cell adhesion and archaeal attachment to the surface (Thoma et al. 2008; Fink et al. 2023). The rest of the archaea were predicted to possess type IV pilins resembling structures (Szabó et al. 2017) or fimbria (Thoma et al. 2008) that might be involved in the surface-associated biofilm formation or intraspecies auto-aggregation. However, exact structure of these archaeal pili and function was identified only for a few currently genetically accessible archaeal genera: *Sulfolobus*, *Methanococcus*, and *Haloferax* (Pohlschroder and Esquivel 2015). Microscopic observations of *Sulfolobus* surface-attached biofilms demonstrated that N-acetylglucosamine-containing type IV pili formed cell–cell connection in the mature EPS-coated biofilms (Koerdt et al. 2010).

#### Archaella/pili

The abovementioned archaeal structures taking role in the surface attachment are different from the motility-associated structures that are anchored to the cell envelope of archaea (archaella) and bacteria (flagella, pili) (Jarrell and Albers 2012). In bacteria, post-transcriptional regulation of the switch between activity of the two cell appendages is known to be regulated by the intracellular concentrations of a second-messenger molecule, like bis-(3′-5′)-cyclic dimeric guanosine monophosphate (c-di-GMP) (Wolfe and Visick 2008; Martinez and Vadyvaloo 2014; Bordeleau et al. 2015)). Free-living non-aggregated bacteria, as well as the ones dispersing from the biofilms, have lower cytoplasmic concentrations of c-di-GMP, compared to the aggregated ones (Poulin and Kuperman 2021; Manner et al. 2023). In archaea, which do not have c-di-GMP, c-di-AMP is instead proposed to take on the regulatory role for the progression of cell cycle, although physiological studies testing this are still in their infancy (Yin et al. 2020). Transcriptome-based studies of free-living and biofilm forming archaea revealed a differential expression of 56 genes (for *H. salinarum*) that might be involved in regulating the switch between motile and sessile lifestyles. Therefore, regulation of biofilm formation in archaea is hypothesized to be many-layered and stricter, compared to that of bacteria (van Wolferen et al. 2018). Observations of a broad range of differentially expressed genes in the early and late-aggregated co-cultures of propionate-oxidizing *S. fumaroxidans* and hydrogenotrophic methanogen *M. hungatei* seems to support this notion (Doloman et al. 2024a). Up to 160 genes were differentially upregulated in the transcriptomes of the methanogen when grown in the year-long aggregated mode with the syntrophic bacteria. While *M. hungatei* archaella was constitutively highly expressed throughout the co-cultivation, archaeal type IV pili were significantly overexpressed in the mature year-old aggregates. On the contrary, the syntrophic bacterial partner, *S. fumaroxidans,* had an upregulated expression of the whole pili operon in the early aggregates, but not in the matured aggregates. This suggests that bacterial pili were more important for the initial establishment of the aggregates, while archaeal type IV pili were needed for the maintenance and stabilization of the matured aggregates. Transcriptomic profiling of the aggregated anaerobic methane–oxidizing microbial communities (50% Ca. *Methanophagales* (ANME-1c) and 20% *Ca. Thermodesulfobacterium*) revealed a similar picture (Benito Merino et al. 2022). Cell appendages of sulfate-reducing bacteria (pili) and methane-oxidizing archaea (archaella) were highly expressed in the aggregates of 300-day-old enrichments.

#### Bacterial flagella

Bacterial flagella may also play a similar aggregate-establishing role in other syntrophic hydrogen-exchanging co-cultures. In studies of butyrate oxidizing *Syntrophomonas wolfei* and *Desulfovibrio alaskensis* co-cultures, *D. alaskensis* required functional flagella to establish aggregates (Krumholz et al. 2015). Microscopic observations of the aggregates of *Methanothermobacter thermautotrophicus* and a propionate-oxidizing bacterium *Pelotomaculum thermopropionicum* revealed presence of flagellum-like filaments connecting the partnering cells and being responsible for the stability of the aggregates. Recombinantly produced flagellin subunits of *P. thermopropionicum* were specifically adhering only to the known syntrophic partners of this bacterium, *M. thermautotrophicus* and *Methanosaeta thermophila* (Shimoyama et al. 2009)*.* Moreover, presence of *P. thermopropionicum* flagellin alone triggered higher expression of *M. thermautotrophicus* genes encoding methanogenesis enzymes, adenosine triphosphate synthase, and hydrogenases. This hints on the involvement of the yet-to-be-identified signal transduction system in the methanogens that recognizes presence of its syntrophic bacterial partner.

In parallel or following the initial cell–cell recognition through adhesins or cellular appendages (archaealla, flagella and pili), *intercellular signaling systems* are involved in the regulation of aggregate formation. Such signaling systems involve combined action of two-component signal transduction systems, sigma factors, and sRNA, and can be dependent on the cell densities of the co-aggregating partners (Condinho et al. 2023). The latter are commonly referred to as “quorum sensing” and are mediated by the signaling molecules such as N-acylhomoserine lactones (AHLs), autoinducers (AI-2), diffusible signal factors, and small–signaling peptides (Papenfort and Bassler 2016). These signaling molecules are synthesized inside the cells and are secreted extracellularly, where upon reaching a certain concentration threshold induce transcriptional changes in the signal receiving cells leading to the biofilm formation/maturation. Signaling cascade regulating biofilm formation has been extensively studied for (pure) bacterial cultures, especially in the context of pathogenic biofilms (Mukherjee and Bassler 2019; Wang et al. 2022). Depending on the level of microbial community complexity, different concentrations of varying signaling molecules can be dynamically controlling the biofilm formation. It remains to be revealed whether biofilm formation in archaea uses quorum sensing systems similar to bacterial. For now, only a few studies report presence of higher quantities of AHL-homologues in the biofilm-forming archaeal cultures (*Methanosaeta*, *Halorubrum*) (Zhang et al. 2012; Liao et al. 2016) or syntrophic bacteria-archaea co-aggregates (Doloman et al. 2024a). In mixed engineered systems, where granular sludge is formed, studies report positive correlation between the increased extracellular concentrations of AHLs and granulation (Ma et al. 2018; Zhang et al. 2020). However, the time-resolved biochemical relationship between the AHL producing and “sensing” microbial groups is yet to be identified.

### The role of biofilm matrix components in aggregate maturation step

After initial partner recognition and cell attachment, mixed bacteria-archaea aggregates continue multiplying within the newly formed biofilm matrix. The matrix itself is comprised of EPS (polysaccharides, (glycol)proteins (glyco)lipids), wired mesh of pili (often electrically conductive), and can embed extracellular DNA, extracellular membrane vesicles (MV), and remnants of S-layer proteins (Karygianni et al. 2020; Flemming et al. 2023; Li Wong et al. 2023). One might, however, argue that production of EPS, eDNA, MVs, and establishment of direct electron transfer (DIET) between the partnering co-aggregated cells is all occurring simultaneously during the initial partner recognition and cell–cell attachment. The reason that the argument is persistent in the field is due to the intrinsic plasticity of the intracellular signaling cascades regulating the cell functions. Moreover, secretion of eDNA and MVs (often containing proteins, nucleic acids, and water non-soluble signaling molecules, like AHLs) has been also reported to occur in dispersed non-aggregating cultures of both bacteria and archaea (Toyofuku et al. 2019; Rumbaugh and Sauer 2020; Mills et al. 2024). And while for now there is no information on the eDNA and MVs secretion in mixed culture bacteria-archaea aggregates, regulation of EPS production and presence of DIET has gotten an increased attention over the last 5 years.

The main component of aggregate matrix, EPS, can contribute up to 90% of the biofilm mass (Fulaz et al. 2019) and plays an important role of keeping the cells arranged within the aggregate. Decades of work on the model biofilm-forming *Pseudomonas* and *Vibrio* species revealed the regulation mechanisms of EPS secretion that are proven to be universally true to the other microorganisms, although forming non-attached aggregates. In the model surface-attached biofilms, production and secretion of EPS involves modulation of the gradients of concentration of c-di-GMP (O’Toole and Wong 2016). High amounts of this ubiquitous bacterial second-messenger molecule (also involved in tuning in the activity of the bacterial flagella and pili) activate biosynthesis of EPS and subsequent formation of a biofilm matrix. The levels of c-di-GMP in the cell are fluctuating based on the environmental stressors exerted upon the cell membrane and resulting activity of the sigma factors and sRNA. As a result, bacterial cell can modulate its response to the outside cues and upregulate biosynthesis of EPS that in turn act as a protective coating for the stressed cells. In the studies of mixed-culture aggregates like aerobic and anammox granular sludge, increased concentrations of c-di-GMP were correlated with the increased amounts of biofilms-associated EPS (both polysaccharide and protein fractions) (Wan et al. 2013; Zhang et al. 2022). In the analysis of the transcriptome from the early- and late-aggregated co-cultures of syntrophic propionate oxidizing *S. fumaroxidans* and hydrogenotrophic methanogens (*M. formicicum* or *M. hungatei*), the syntroph demonstrated reliance on the c-di-GMP cycling to regulate its polysaccharide secretion (Doloman et al. 2024a). Half of the syntroph’s c-di-GMP synthesis-associated genes (diguanylate cyclases) were localized in the operons with other signal transduction genes (CheY, PAS/PAC), and were upregulated in the early aggregates, at the onset of macroaggregates formation. The other diguanylate cyclases were localized in the operons with polysaccharide biosynthesis genes (biosynthesis of UDP-GlcNAc, GDP-L-fucose, and polysaccharide assembly/transport) and were among the highly expressed genes in both aggregate initiation and maturation stages. The genes for c-di-GMP-recycling/hydrolysis (phosphodiesterases) were explicitly upregulated in the late-aggregates, suggesting that *S. fumaroxidans* was aiming to maintain the low levels of c-di-GMP in the cells that start dispersion from the biofilm aggregates.

Biosynthesis of EPS in mixed communities might be promoted by the cross-feeding of the EPS precursors (nucleotide sugars) between the co-aggregating partners. Analysis of the transcriptome of activated sludge–derived photogranule revealed a metabolic interaction between phototrophic cyanobacteria *Oscillatoriales* and filamentous bacteria *Chloroflexi* (Kong et al. 2023). The first was proposed to supply nucleotide sugars (UDP-GlcNac, UDP-Glc) to the latter, which were subsequently used to synthesize the exocellular polysaccharides. While production and extracellular transport of nucleotide sugars is also reported for other biofilm-forming microbial communities (Fritts et al. 2021), it is not a trivial task to identify the subsequent fate of these EPS-precursors in the co-aggregating partners. Knowledge of EPS biosynthesis pathways is currently restricted to aerobic microorganisms, with confirmed genes involved in the biosynthesis of glycans such as alginate, PNAG, cellulose, curdlan, diutan, salecan, succinoglycan, and xanthan (Whitfield et al. 2020; Dueholm et al. 2023). Meanwhile, microorganisms in anaerobic communities, especially archaea, lack those EPS-biosynthesis genes, or have truncated operons (Doloman et al. 2024b). From the limited studies on *Sulfolobales* species, archaeal EPS are found to contain interlinked repeats of glucose, galactose, mannose, and *N*-acetyl-D-glucosamine (Kuschmierz et al. 2022). Investigations on the structural composition of EPS in mixed bacteria-archaea aggregates (anammox and anaerobic granular sludges) revealed presence of a great diversity of pentoses, hexoses, and N-acetylhexosamines (Boleij et al. 2018; Doloman et al. 2024b). Yet, studies addressing the biochemical pathways that are associated with biosynthesis of these glycans are scarce, and it remains to be seen how microorganisms in these mixed communities exchange EPS precursors.

Within the EPS matrix, minor components of biofilms, like nanotubes and cellular appendages, mediate aggregate stability. While the importance of electron exchange within the mixed bacteria-archaea aggregates has been elaborated in “A nutritional need to cooperate” (often via exchange of H_2_ or formate), direct electron transfer (DIET) can be regarded as a special case for such exchange. DIET occurs via cell nanowires, often represented by electrically conductive pili or archaella (e-pili/e-archaella). Bacteria predicted to possess these e-appendages (Walker et al. 2018) have been observed to have tendency to aggregate with their DIET partners. Co-aggregation of ethanol oxidizing bacteria and methanogens (*Geobacter* and *Methanosarcina*/*Methanosaeta*) is the best example, where DIET takes over conventional extracellular H_2_ and formate exchange (Rotaru et al. 2014; Yee and Rotaru 2020; Zheng et al. 2020; Holmes et al. 2022). Analysis of the metatranscriptome in methanotrophic aggregates and anammox granules pointed to the involvement of bacterial type IV pili/flagella and archaeal multiheme cytochrome c-like proteins in electron transfer within the aggregates (Wegener et al. 2015; Benito Merino et al. 2022). However, experimental proof for the presence of DIET in syntrophic anaerobic aggregates is yet to be obtained.

**Fig. 3:**
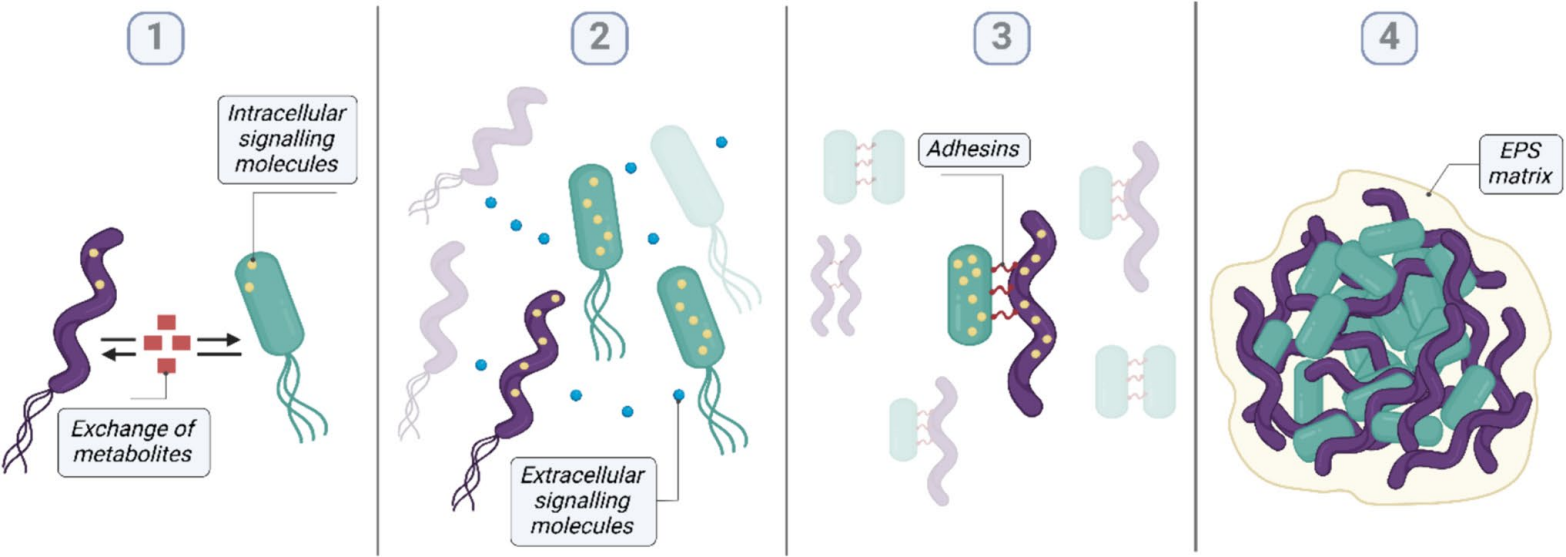
A 4-step conceptual model of co-aggregation in mixed anaerobic cultures which proceeds through: (1) mutualistic metabolic interactions, (2) population growth and extracellular signaling, (3) “first contact” and loss of motility, (4) aggregate maturation by expansion of EPS matrixome. Detailed description of the steps can be found in the text above. Illustration created with BioRender.com

Summarizing the available knowledge on the multi-species aggregates, we propose a 4-step process of mixed anaerobic bacteria-archaea co-aggregation (Fig. 3). Step 1 depicts the initial microbial community in the suspended/planktonic mode, which is low in cell density and is comprised of motile cells that are actively moving in the environment and exchanging gaseous and/or liquid-soluble metabolites. In this state, both bacterial and archaeal partners have low levels of the intracellular signaling molecules (like c-di-GMP or c-di-AMP). As the population cell density grows, so do the levels of signaling molecules, while some also diffuse extracellularly or are actively transported as a cargo in membrane vesicles (step 2). In parallel to this, particularly between the cells that are located closer to each other, co-aggregation begins with initial cell–cell contact (step 3). Upon the first contact, cells produce attachment-mediating adhesins. Simultaneously, because of the continued accumulation of the signaling molecules, co-aggregated cells halt expression of movement-associated cellular appendages (e.g., flagella) and become static. The resulting irreversible cell-to-cell attachment and stronger pili and adhesin-mediated connections lead to further alterations in the cellular metabolism of both partners. Increased quantities of the signaling molecules activate production and secretion of EPS, promoting even stronger aggregates (step 4). Aggregates of cells glued by EPS continue exchanging metabolic and signaling cargo until the diffusion limitations of the aggregate are reached. Although not shown in Fig. 3, diffusion limitation leads to the development of zones with dead biomass within the aggregate that can in turn result in the weakening and eventual breakage of the cell clumps into smaller ones. Smaller clusters can continue to grow by expansion or by incorporating new members from outside.

## Future directions and conclusions

While it is clear that mixed anaerobic microbial communities often utilize cellular appendages similar to those found in aerobic microorganisms (flagella, pili) to form and stabilize the aggregates, the biochemical signaling cascade that regulates these protrusions remains to be understood. The fact that the majority of the anaerobic aggregates are comprised of nutritionally dependent symbionts and even strictly obligate syntrophs (Fig. 2) makes it challenging to separate one bacterium/archaeon from the community to study its aggregation-associated physiology in the right ecological context. A possible solution lies in selectively genetically engineering one of the co-aggregating partners (for instance, altering the cellular appendages system) and assessing the effect of the modifications on the overall stability of the co-aggregating community. Recent advancements in the application of the CRISPR Cas systems on diverse methanogenic archaea can allow to finally bring the research on anaerobic co-aggregation to the level of mechanistic understanding that is so rich in the aerobic biofilm studies (Bao et al. 2022; Li et al. 2022).

To advance studies of anaerobic aggregates in mixed culture mode, more detailed species tracking technologies need to be developed and tested. For example, development of a real-time imaging system that allows to track microbial aggregation in anoxic conditions would require a far more advanced laboratory infrastructure, compared to the one needed to track aerobic biofilm formation (Chia et al. 2020; Hartmann et al. 2021), e.g., requiring oxygen-independent fluorescent tagging systems (Flaiz et al. 2022) and anaerobic chambers equipped with microscopy and microfluidic growth chambers. Currently, there are a few studies reporting successful implementation of anoxic microfluidic and flow cell set-ups that aid investigating the short-term (48 h) bacterial growth and attachment (García-Bayona et al. 2020; Wang et al. 2013). However, there are no reports yet for the continuous monitoring of the anoxic microbial growth for a prolonged time (e.g., months) that is the necessary to track formation of mm-sized anoxic aggregates, like the ones of syntrophic propionate–oxidizing bacteria and methanogenic archaea (Doloman et al. 2024a). Similarly, high-throughput screening of the co-aggregating partners from the strictly anoxic and often metabolically depended microbial communities would enable to systematically assess the role of nutritional cooperation in promoting anaerobic aggregation (Huang et al. 2023). Time-resolved metabolite tracking studies in these complex communities can be useful in directing the more detailed bi- and tri-culture studies that can still preserve nutritional interdependencies yet are less complex than the natural co-aggregating associations (Doloman et al. 2022, 2024a; Besteman et al. 2024). Since all the known anaerobic aggregates (performing anaerobic digestion, anammox or anaerobic methane oxidation) do show a significant degree of syntrophic relationships between its members, it would be curious to see whether syntrophy is indeed the preferred lifestyle in anoxic environments. Eventually, insights into these obligatory nutritional cooperations can have interesting implications for studies of evolutionary microbiology and development of early life on Earth, where oxygen was definitely a rare electron acceptor. Is “selection of the fittest” only applicable to the oxygenic world, and anoxic early Earth was a place thriving instead with cooperations?

Advancements in the microbiological and molecular techniques of the next years are yet to allow the researchers to uncover the full spectrum for applications of microbial aggregates to achieve a sustainable human existence (Philipp et al. 2023). Understanding the community dynamics and cellular crosstalk that governs anoxic aggregation will not only stabilize application of the biotechnologies already in use (like biological wastewater treatment), but also broaden industrial applications of the anoxic microbial communities as biocatalysts for production of specialty biochemical commodities, like pharmaceuticals and nutraceuticals.
